# Valuing life and evaluating suffering in infants with life-limiting illness

**DOI:** 10.1007/s11017-020-09532-x

**Published:** 2020-12-17

**Authors:** Dominic Wilkinson, Amir Zayegh

**Affiliations:** 1 Oxford Uehiro Centre for Practical Ethics, Faculty of Philosophy, University of Oxford, Oxford, UK; 2 Wellcome Centre for Ethics and Humanities, University of Oxford, Oxford, UK; 3 John Radcliffe Hospital, Oxford, UK; 4 Murdoch Children’s Research Institute, Melbourne, VIC Australia

**Keywords:** Infancy, Newborns, Value of life, Quality of life, Withholding treatment, Cognition disorders

## Abstract

In this paper, we explore three separate questions that are relevant to assessing the prudential value of life in infants with severe life-limiting illness. First, what is the value or disvalue of a short life? Is it in the interests of a child to save her life if she will nevertheless die in infancy or very early childhood? Second, how does profound cognitive impairment affect the balance of positives and negatives in a child’s future life? Third, if the life of a child with life-limiting illness is prolonged, how much suffering will she experience and can any of it be alleviated? Is there a risk that negative experiences for such a child (suffering) will remain despite the provision of palliative care? We argue that both the subjective and objective components of well-being for children could be greatly reduced if they are anticipated to have a short life that is affected by profound cognitive impairment. This does not mean that their overall well-being will be negative, but rather that there may be a higher risk of negative overall well-being if they are expected to experience pain, discomfort, or distress. Furthermore, we point to some of the practical limitations of therapies aimed at relieving suffering, such that there is a risk that suffering will go partially or completely unrelieved. Taken together, these considerations imply that some life-prolonging treatments are not in the best interests of infants with severe life-limiting illness.

## Introduction

Cases like that of baby Esther, who has severe lissencephaly and necrotic bowel, raise extremely difficult ethical questions.^1^ Esther’s future life is likely to be short, it is certain that she will have profound neurological impairment, and she has a potential for significant suffering. Is it in her best interests to have her life prolonged by surgery? Would that life be worth living?

Answering such questions is challenging because of the epistemic problem of evaluating an infant’s subjective experience. It is also challenging because of the philosophical difficulty of deciding on, evaluating, and weighing the different components of well-being in a child’s current and future life. These formidable problems sometimes lead professionals and ethicists to argue that such questions are unanswerable and, therefore, that parents’ wishes should be decisive.

In this paper, we explore three separate questions that are relevant to assessing the value of life for infants like Esther: (1) What is the value or disvalue of a short life? Is it in the best interests of a child to save her life if she will nevertheless die in infancy or very early childhood? (2) How does profound cognitive impairment affect the balance of positives and negatives in a child’s future life? (3) If the life of a cognitively impaired child with life-limiting illness is prolonged, how much suffering will she experience and can any of it be alleviated? Is there a risk that negative experiences for such a child (suffering) will remain despite the provision of palliative care? We will address these questions individually and sequentially, though their respective answers are interrelated. Our aim is not to definitively settle the question of treatment for Esther. Nevertheless, the answers we give may be useful to parents and professionals trying to decide whether or not to embark on surgery.

For the sake of this analysis, we will set aside some of the empirical uncertainties that often make clinical decision-making difficult—for example, the extent of future disability and length of potential lifespan. We assume that in this case it is certain both that Esther will have profound neurological impairment such that her maximum cognitive capacity will be that of an infant and that she will die in childhood.^2^ We also focus on Esther’s prudential well-being—that is, how well or badly her life will go for *her*.^3^ We will not discuss in any detail separate relevant considerations pertaining to the interests of others or to the value of Esther’s life for others—for example, the interests of her parents or the wider society or issues relating to distributive justice. We are not assuming a particular theory of well-being for this analysis [4], though in parts of the paper we compare the implications of hedonic and objective list theories.^4^ Instead, we draw on the intuitive idea that assessment of Esther’s overall well-being (and best interests) reflects a balancing of positives and negatives in her current and future life—a notion we refer to as ‘net well-being’ [5]. Finally, we use the term *suffering* to refer to negative hedonic states. On objective views, there may be some negative elements of well-being that are not directly experienced [6]. We believe that these may be relevant to decision-making but are conceptually distinct, and we will not discuss them in this paper.

## What is the value or disvalue of a very short life?

One element of Esther’s prognosis is the expectation that she will die in childhood. An obvious implication is that she has a reduced amount of well-being to look forward to. However, the amount of well-being in a life is not likely to be evenly distributed across a lifespan. Some periods may have more well-being; others may have less. There may even be periods (taken alone) that have negative net well-being. What is the prudential value of the first phase of life?

Before answering that question, we should separate out the value of life from the disvalue of death. On one plausible view, death is bad for us at least in part because it deprives us of future well-being [7]. Premature death is particularly bad because of the shortened length of future life and because of the amount of positive well-being that is thereby lost. Yet our focus here is different. We are assessing how good it would be to live, not how bad it would be to die. When we are thinking about the value of a life, the absence of a period of positive well-being (a year of happiness, say) represents a reduction in what we have to consider on the plus side of the scales of well-being. It does not itself constitute a negative, as that would double-count the loss.^5^

We could consider first the very shortest of lives. Imagine that you had been stillborn in late gestation or during the birth process. Would you have had a life worth living? Would it have been better for you to have never been conceived?^6^

Potentially, life in utero has no prudential value—either positive or negative. In terms of subjective well-being, there are difficult questions about whether and when foetuses are capable of experiencing pain. However, foetuses are not likely to be cold or hungry, and in the normal course of in utero development, there would not be any aversive stimuli likely to cause pain. There are similar epistemic problems in knowing what positive experiences foetuses may have. They may experience warmth and physical containment, but would lack most of the stimuli that appear to soothe the ex utero infant, such as feeding, holding, caressing, and comforting.

Does foetal life contain any objective value? As with most questions related to objective elements of well-being, the answer to that question will depend on which particular set of objective values are included. However, most lists include components that are dependent on consciousness [9]. On such theories, many, if not all, elements of well-being might be assumed to be absent during foetal life.^7^

One possibility is that foetal life is something like a sleep state.^8^ It might be thought that sleep, as a period of unconsciousness, has zero contemporaneous contribution to well-being, but rather gains its value upon waking due to the instrumental value of a state of rest as well as the retrospective appreciation that one might feel for having slept. Foetal life may similarly gain its value once the foetus is born. But, ultimately, it is difficult to reach any confident conclusions about the prudential value of foetal life.

What of an infant who dies early? Consider, for example, an otherwise normal infant who dies from sudden infant death syndrome at six weeks of age. What is the value of that short life for such an individual?

On one view, very short lives might be intrinsically bad. For example, David Benatar argues that even full-length human lives are frequently bad [11, ch. 3]. He cites the negative mental states, preference frustration, and lack of objective value that characterise almost all human life [11, pp. 69–86]. Benatar’s view might appear extremely pessimistic, and many do not share it. Nevertheless, even if one is not inclined to accept the radical conclusion that there is negative net well-being in a full-length human life, some of Benatar’s insights might be relevant to an assessment of very short lives. Benatar lists some of the common negative hedonic states that afflict all of us on a daily basis: ‘hunger, thirst, bowel and bladder distension (as these organs become filled), tiredness, stress, thermal discomfort (that is, feeling either too hot or too cold), and itch’ [ 11, p. 71]. He suggests that significant portions of each day are marked by one or another of these states and that their pervasiveness is often underestimated. Relief from these states sometimes leads to physical pleasure—such as pleasure from eating or the transient positive sensation from scratching an itch—but more commonly simply returns us to a neutral (or preexisting) hedonic state.

These common negative sensations certainly affect infants. However, as parents of newborns will freely attest, the ever-present challenge is in knowing what kind of sensation is present at any particular moment. Is the infant crying because she is still hungry or, perhaps, because she has overeaten and is experiencing gastroesophageal reflux? Is she uncomfortable and in need of soothing, or is she tired and in need of sleep? The challenge for parents, especially new parents, is that it can be extremely difficult to distinguish between different causes or types of distress. What is more, the distress of newborn infants is often intense and protracted. From one to three months of age, infants reportedly cry an average of two hours per day, with some infants experiencing considerably longer periods of crying, particularly around the four to six week mark; and ‘unsettled’ infants referred to health professionals have been reported to cry for more than five hours per day [12]. No one can know the subjective experience of an infant. Crying behaviour obviously has an important functional and evolutionary role, and it is possible that at least some infant crying is not indicative of a strongly negative mental state. Yet it would be a mistake to discount infant crying for that reason. The external evidence indicates a state of intense anguish. There may not be a parallel mental state, but such intense manifestations in an older person would usually be seen only in the presence of severe physical or mental pain. One should be very loath to ignore that evidence, while also acknowledging that it is difficult to know just how bad the corresponding states are.

An important and related question is whether states of distress in infants have an affective dimension that is equivalent to states of distress in older children or adults. Philosophical and scientific studies of pain have often distinguished between sensory and affective dimensions [13]. It has long been thought that much of the badness of pain is its affective element—this is, the quality of the pain experience that leads us to seek to avoid it [14]. If the affective element of infant pain (or distress) were diminished or absent, then the negative value of pain in infancy would be considerably less than that of similar states later in life. There are two ways of responding to this claim. The first is to emphasise the fundamental difficulty of assessing the affective dimension of a subject’s experience when the subject is unable to report it. One simply cannot know what pain or distress is like for an infant, and one’s assessments are liable to be influenced by one’s own empathy for the infant [15]. The second is to note that functional neuroimaging studies of infants experiencing painful stimuli indicate patterns of activation that are very similar to those seen in adults [16]. This may imply that the affective dimension of painful experience (and the unpleasantness) in infants is also similar to that in adults.

Are there correlative positives or pleasures in the lives of newborns? Here, too, their developmental stage makes it difficult to be certain. Newborns certainly experience relief from negative sensations, and they also appear to appreciate some simple pleasures—for example, from physical touch and the sensation of being held or carried or from visual, olfactory, and auditory signs of the proximity of their mother. Outward signs of pleasure are limited in very young infants (smiling typically emerges at six weeks of age). Of course, a lack of positive cues does not mean a lack of positive experiences. However, it is hard to identify any positive experiences in newborns that are as intense as the corresponding periods of negative well-being.

What of other theories of well-being? Preference- or desire-based accounts do not offer a straightforward way of evaluating newborn life. Newborns clearly have some preferences, such as relief from the negative physical sensations described above, as well as desires for attention and the proximity of parents. Yet it is difficult to make any meaningful assessment of the degree to which an infant’s preferences are satisfied or frustrated. Do objective list theories fare any better? Many of the elements that are often listed as being objectively valuable for human life are not ones that are accessible or available to the infant. One element, development of deep interpersonal relationships, is clearly present for most newborns. Indeed, the intensity of the parent–infant bond developed at this stage might mean that this value is highly relevant, and positive, for the infant. Yet it is harder to identify other objective values that might apply.

While there are certainly some positives in the lives of newborns, arguably the principal value of life lies ahead of the infant. This value arises both from an infant’s future subjective experience and from the realization of future objectively valuable elements of well-being, such as agency, understanding, enjoyment, learning, and the development of broader interpersonal relationships.

It is not clear how to weigh up the overall well-being of infant life. Perhaps the negative physical sensations and distress must simply be endured for the sake of the positive well-being to come (certainly, some parents entertain this sort of sentiment during the initial exhausting period of caring for a newborn). Or perhaps the value of the physical consolations and developing parent–infant bond outweighs the periods of crying and distress. There are no easy answers.

Given the discussion above, how should one approach the case at hand? Personally, we are agnostic about the overall prudential value of life for an otherwise healthy infant who dies suddenly at six weeks of age. Nevertheless, we contend that the value is at best mildly positive. It seems implausible to us that the life of such an infant would be strongly positive for her. This intuition is important because if there is reason to believe that an infant who will have a short life will suffer more than average, then there may be reason to think that her short life would not be worth living. If, for example, it is known that Esther will die at six weeks of age, there is a strong case to be made that surgery would not be in her best interests. Of course, this will depend on how much suffering Esther would experience as a consequence of the surgery. If she had perfect analgesia, such that she would experience no pain from surgery, that argument may not pertain. (We return below to the problem of perfect analgesia and reasons why it may not be achieved.)

Though a more in-depth discussion is beyond the scope of this paper, in our view, the outcome that has the most reason to render a life not worth living is not survival with long-term disability, but death after a prolonged period in intensive care. An extremely premature infant who dies after a tumultuous four-month period in neonatal intensive care has unquestionably experienced negative net well-being. The infant will have experienced little in the way of positives, and it does not seem conceivable that those positives would outweigh the negatives. It may have been reasonable to embark on provision of neonatal intensive care because of the infant’s uncertain outcome and the possibility that the infant might survive to enjoy a life worth living. However, if it were known in advance that death was the certain outcome, we suggest that it would have unquestionably been unethical to initiate life-sustaining therapy.

If we are right that very early human life contains relatively less overall well-being, how long does this extend? Is it confined to the first months of life, or does it stretch to the second year of life or later? We are going to have to set aside such questions for this paper. Suffice it to say that early life contains fewer elements of well-being than later life in large part because of the developmental stage of newborns and infants. This relation between developmental stage and reduced well-being may be particularly relevant when considering individuals whose long-term capacities will never advance beyond those of an infant.

## How does profound cognitive impairment impact on well-being?

In Esther’s case, her profound neurological developmental disorder will, we contend, unquestionably affect her future well-being. However, how it affects her well-being, and to what extent, will depend upon the theory of well-being that is applied as well as her specific circumstances.

One obvious way that profound cognitive impairment affects well-being is through its effect on a child’s ability to realise objective goods—elements of well-being that are regarded (by some at least) as constitutive components of human flourishing. Conditions or disabilities that lead to loss of capacities or functions do not always cause net reductions in well-being, since there are many different ways of achieving a fulfilling life. As Adrienne Asch and David Wasserman point out, ‘human beings enjoy a fortunate redundancy in many of the capacities that are instrumental for, or constitutive of, valuable human goods and activities’ [17, p. 208]. However, some forms of disability and some degrees of disability can make it extremely difficult if not impossible to attain valuable human goods or engage in valuable human activities.^9^

For example, James Griffin lists accomplishment, agency, understanding, enjoyment, and deep interpersonal relationships as constituents of well-being [19, p. 67]. It appears that Esther may have no prospect of accomplishment, agency, or understanding, although she may remain capable of enjoyment and deep interpersonal relationships.^10^ On other accounts of well-being, there may be more elements potentially missing for Esther. Derek Parfit lists ‘moral goodness, rational activity, the development of one’s abilities, having children and being a good parent, knowledge, and the awareness of true beauty’ [8, p. 499], all of which will potentially be absent for Esther.^11^

The relevance of Esther’s impairment here is its likelihood of reducing positive objective elements of well-being in her future life. Like a reduced lifespan, reduced well-being should be thought of as an absence of or reduction in benefits. These absences do not constitute negative well-being, though they may mean that negatives more easily outweigh positives and may render her more likely to have negative net well-being.

However, Esther’s neurological disorder may also affect her positive and negative subjective experiences. In what follows, we present three different views about the effect of profound cognitive impairment on subjective experience: positives and negatives attenuate symmetrically, negatives attenuate more yielding a net benefit, or positives attenuate more yielding a net reduction.

### Symmetrical attenuation

One possibility is that severe neurological disorders causing profound cognitive impairment reduce positive and negative experiences symmetrically. A child who has reduced awareness might miss out on certain experiences that would have caused pleasure, but she might also miss out on certain things that would have caused sadness or displeasure. As an example, children with profound cognitive impairment will almost certainly never experience the pleasure of romantic love, but they will also never experience the anguish of a broken heart or unrequited love.

One could think of this attenuative process as analogous to the volume control on a stereo or radio. Turning down the volume by rotating the dial reduces both pleasant and unpleasant sounds—and it potentially does so to an equal extent. If assessments of overall well-being reflect the balance between positives and negatives, then the net effect of cognitive impairment on a child’s subjective well-being would be, on this view, neutral. To take another perspective: if one imagines a random group of children and considers their overall well-being, some will be very happy, having many more positive than negative experiences, others will be the opposite, and many will be somewhere in the middle, having an average mix of positive and negative experiences. If one now considers a group of children who are profoundly cognitively impaired, but with otherwise identical features, the distribution of subjective well-being would be identical on this view.

### Asymmetrical attenuation—net benefit

In terms of subjective well-being, it would be very good if profound cognitive impairment had no net effect. Then parents of children like Esther could find reassurance in the knowledge that their child is no more likely to be unhappy than any other child. However, it seems somewhat implausible that this would be the case. Severe neurological disorders causing profound cognitive impairment radically change the things that a child will experience as well as the way in which she will experience them. Their effect on the overall balance of subjective well-being might be neutral, but one should not simply assume this to be true.

One possibility is that reduction in cognitive capacity eliminates some sources of distress or negative experience while allowing children to continue to experience positives. In particular, children might be less likely to be aware of themselves and the things that they are unable to achieve. They might also be less likely to undergo certain types of negative experience, such as anticipatory anxiety. Reduced cognitive capacity might then have a net *positive* effect on subjective well-being.

Elsewhere, one of us has described a form of this phenomenon as the ‘tolerability paradox’ [22]. In the setting of progressive cognitive decline, adults with dementia can experience a phase during which they are distressed by the loss of capacities and abilities that they formerly took for granted and potentially depressed and anxious at the prospect of what lies ahead for them and their families. However, as their dementia deepens, these sources of distress may disappear. With loss of awareness, their subjective experience may become more tolerable, even as their condition worsens.^12^

It seems possible that children with severe cognitive impairment might be happier overall (or more likely to be happy) than children with milder degrees of cognitive impairment. If that were reliably the case, parents of children diagnosed with severe brain disorders would perhaps have even more reason to be consoled than they would on the symmetrical attenuation view.^13^

### Asymmetrical attenuation—net reduction

While profound cognitive impairment could have a net positive effect on well-being, the opposite possibility should also be considered.

One reason for thinking that the balance of subjective well-being might be tipped in a negative direction relates to the arguments that we developed in the first section of this paper, where we suggest that the neonatal period and early infancy contain more negatives and fewer positives relative to later life stages. The negative subjective experiences of newborns can be intense and prolonged, and they may have fewer or less intense counterbalancing pleasures. Some may believe, for this reason, that a newborn who dies at six weeks of age has had a life of negative net well-being, or (less controversially) has experienced less well-being than she would have in a corresponding six-week period later in life.

If this belief is correct, then it would also potentially apply to children whose cognitive capacities remain at the level of a newborn or early infant.^14^ Indeed, there are some children, and Esther may fall within this category, who will never develop beyond this early developmental stage. The concern may be all the greater for some neurologically impaired children who manifest extreme cerebral irritability [25]. In such children, ordinary stimuli may (or may appear to) lead to substantial distress, and it seems plausible that their overall well-being would be negative.

However, whether or not the above arguments about the subjective well-being of newborn infants hold, it is necessary to consider the situation of children whose cognitive capacities and awareness may be considerably lower, even, than a newborn. Some children may have only minimal levels of consciousness or awareness throughout their life. What effect would such impairment have on overall well-being?

We offered an analogy earlier, suggesting that cognitive impairment might diminish experiences similar to how the volume dial on a stereo reduces sounds. However, turning down the volume on a stereo does not always reduce positive and negative sounds equally. Sometimes unpleasant sounds are louder or more acoustically salient than more pleasant or interesting ones. Many will have had the experience of a radio whose volume is turned down sufficiently low that it is not possible to follow conversations or hear melodies, but some unpleasant or annoying sounds can still be perceived. Could there be anything similar for conscious experience? It is, of course, epistemically challenging to know what life is like for those who are minimally conscious. We suggest, however, that the experience of physical pain and discomfort is one of the most basic neural sensations. As such, physical pain and discomfort could potentially be present even at extremely low levels of consciousness. The corresponding positive basic sensations (perhaps satiety, comfort, or physical pleasure) might require a higher level of awareness.

A comparison can be drawn with surgical anaesthesia. The aim of such anaesthesia is obviously to remove sensation (and recall) of pain.^15^ With higher doses and more potent combinations of anaesthetic agents, the anaesthetist induces deeper levels of unconsciousness, reducing the chance that the patient will perceive pain during surgery. In this way, more painful procedures will require more anaesthetic agents. At lighter levels or planes of anaesthesia, it is possible that the patient will perceive pain [27], but it is far less likely that the patient will experience positive physical sensations.^16^ If this comparison holds, it might apply, for example, to patients in a minimally conscious state. Disorders of consciousness exist on a spectrum—coma, persistent vegetative state, minimally conscious state, and severe neurological impairment [28]. Patients with minimal levels of consciousness might be like patients who are partially anaesthetised—they are potentially capable of experiencing physical pain, but incapable of experiencing counterbalancing pleasures.

We could call this claim the dyshedonia hypothesis.^17^

**Dyshedonia hypothesis**. Very low levels of consciousness are associated with overall negative well-being, since in such states it is possible for one to perceive pain and negative sensations but not possible (or less likely) for one to perceive positive experiences.

If this hypothesis is correct, it would be very bad, for example, to be in a minimally conscious state [29].^18^

We have outlined three different views about what might happen to subjective well-being with profound cognitive impairment—symmetrical attenuation, asymmetrical benefit, asymmetrical diminishment. Which of these is correct? The challenge, of course, is that there is no way of definitively answering this question. When it comes to the subjective experience of those with profound cognitive impairment, one can never know what life is like. Some may suspect that such impairment is hedonically neutral; some (who are perhaps more optimistically inclined) may think that it is a positive; and others may take a more negative view informed by the dyshedonia hypothesis.

We cannot know, but here is one proposal: perhaps all three of these views may apply, but at different levels of impairment. Some degrees of cognitive impairment may have no net effect on subjective well-being. At more severe levels of impairment, there may be a relative positive effect on subjective experience. And at the most profound levels (corresponding to minimal consciousness), the dyshedonia effect may predominate.

## How much suffering will be experienced? Is it likely to go unrelieved?

On any of the views that we have described, there may be positive and negative subjective components of well-being. Whichever view is correct regarding the usual balance of well-being for a child like Esther, it is possible that the negatives will outweigh the positives. However, whether this occurs will depend upon how much discomfort, distress, or pain she experiences. If she suffers little or not at all, she will potentially have positive net well-being.

The presence and extent of Esther’s suffering will be determined by both the nature of her condition and its effect on her conscious experience. However, it will also depend on the medical treatment that she receives. If Esther is anticipated to experience a significant degree of suffering during her short life—as a consequence of her illness or as a consequence of medical treatment—one might expect that her suffering could be detected and treated by health professionals. In theory, symptom treatment for Esther could and should relieve symptoms of discomfort or distress. It should be possible to directly treat conditions leading to such symptoms and to titrate symptom-relieving medication to a level that would relieve negative sensations. If necessary, pain could be removed by high doses of analgesia or sedation. Is there reason to think that such treatment would be insufficient and that Esther would nevertheless have unpleasant subjective experiences?

There are at least four factors suggesting that, in practice, children like Esther sometimes experience significant unrelieved suffering: unreliable pain assessment, scientific uncertainty, the effect of analgesia on positive experience, and limitations to analgesia. These factors relate to particular challenges in treating pain in very young children or children with severe neurological impairment. They do not mean that children like Esther will necessarily suffer. Nevertheless, they mean that such children may be more likely to have negative well-being.

### Unreliable pain assessment

First, since pain is by definition a subjective phenomenon, one can make only second-hand assessments of pain and discomfort for infants and patients who cannot communicate expressively. These assessments will be based on a limited repertoire of behavioural and physiologic signs. Their dependence on others to detect and act on limited indirect signs of pain and discomfort means that the suffering of such patients often goes under-recognised.^19^ Children with severe neurological impairment have an even smaller repertoire of behavioural and physiologic signs, so it is all the more difficult to detect when they are suffering (the ‘silent suffering’ problem) and, when they appear to be suffering, to determine the cause.^20^

### Scientific uncertainty

Second, treatments for pain in infants are more uncertain than they are in adults and older children. While most pain medications and their dosing have been extrapolated from adults, there is a paucity of evidence about which analgesics work in these patients and what doses are required to achieve effective analgesia. The lack of evidence is due in part to the ethical and practical challenges of performing drug trials using this vulnerable group [34]. Uncertainty may lead to overtreatment and harm—for example, there is evidence that oral morphine for procedures might cause harmful side effects in infants without necessarily offering effective pain relief [35]. Perhaps more frequently, however, uncertainty (combined with an understandably precautionary approach) may lead to undertreatment of pain in infants.^21^

### Effect of analgesia on positive experience

Third, alleviation of suffering may also diminish the pleasures that infants can experience. For example, in the presence of severe pain and discomfort, high doses of centrally acting analgesics may be required, and these tend to cause significant sedation.

The overall effect of analgesia on the balance of positive and negative experiences in an infant’s life might align with one of the views described above—that is, analgesia might diminish positive and negative experiences symmetrically or asymmetrically. If the effect of analgesia is asymmetrical, it is possible (and certainly desirable) that pain and discomfort would be reduced more than positive experiences. Yet there is also the possibility that at high levels of sedation, and particularly when a child’s baseline conscious experience is already much lower than normal, the dyshedonia effect may predominate. This could lead to a paradoxical situation in which treatment of an infant’s suffering actually removes her positive experiences before it completely removes her negative ones. For infants or children with cognitive impairment, it may take lower doses of analgesics to reach such a dyshedonic state or there may be a higher likelihood that the dyshedonia effect will occur.

### Limitations to analgesia

Finally, the administration of analgesia itself is subject to certain limitations. In theory, if clinicians are concerned that Esther might be suffering despite analgesia, they could use an even higher dose of sedation in order to obliterate any residual suffering that she might be experiencing (while also potentially rendering her completely unconscious). There are, however, a number of reasons why using sedation to eradicate possible suffering may not be achievable or desirable. There are other ethical priorities that may compete with the desire to remove suffering and thereby lead either clinicians or parents (or both) to resist high doses of sedatives and analgesia.

One competing priority is the desire to avoid harm. High doses of sedative or analgesic medications can cause states of physiologic instability, such as low blood pressure [36]. They may also suppress an infant’s respiratory drive. Parents may resist high doses of sedatives and analgesics because of their desire to prolong their child’s life coupled with anxiety about the possible life-shortening effects of these medications. Indeed, it is accepted that, when used at high doses in the context of end-of-life comfort care, sedatives and analgesics may shorten the lives of infants [37]. Their use on infants is nonetheless widely justified as morally permissible by appeal to the doctrine of double effect,^22^ as reflected in the prevalence of such drugs in palliative care medicine. In older children who have life-limiting conditions but are not dying, judicious use of sedatives and analgesics has not been shown to have life-shortening effects [38]; however, anxieties remain about their use.

Another competing priority relates to the value of conscious experience. As noted above, adequately relieving suffering in some patients may require heavy sedation, which necessarily reduces their level of conscious awareness. The capacity for consciousness is widely seen as having moral significance, so much so that life-sustaining treatment is often withdrawn from patients who have irreversibly lost the capacity for consciousness, such as those in a persistent vegetative state or with brainstem death following severe brain injury. While the moral significance of consciousness is beyond the scope of this paper, it suffices to point out that parents may deem it important for their children to have some periods of conscious awareness to at least allow for the possibility of positive interactions with them. At a level of conscious awareness where infants could benefit from interaction with loved ones, they would also have a capacity to feel pain if pain is present. In this way, there may be a conflict between parents’ desire for their child to be conscious and clinicians’ desire for the child not to be in pain.

A further conflicting priority may be the desire to facilitate weaning from respiratory support. When a child is mechanically ventilated, the use of sedatives or analgesics (sometimes introduced so that the child does not experience discomfort from being on the ventilator) may reduce respiratory drive and effort. Either parents or clinicians (or both) may desire for sedation to be reduced in order that the child’s respiratory support can be reduced. This step can be important if the child is to leave intensive care. In patients whose illness does not render them permanently ventilator dependent, facilitating discharge home may be of sufficient importance to aim for lighter sedation, at the expense of total relief of all pain and discomfort.

In view of all of these considerations, we have argued that it is possible that Esther and similar infants will experience unrelieved suffering. This possibility is due both to limitations in the detection and relief of suffering and to competing priorities around the extent to which sedation is justified as a means of reducing suffering. If it is the case that Esther’s suffering is unlikely to be fully alleviated, then this fact would lend further weight to our suspicion that Esther’s prudential well-being is negative. While residual suffering is often tolerated in patients who have a chance of recovery (and therefore a life of positive net well-being), there is less justification for tolerating suffering in patients with severe life-limiting conditions where there is no major uncertainty regarding future outcome. There is even less justification for tolerating suffering in terminally ill patients who have profound cognitive impairment, since such patients have minimal access to the experiential pleasures or objective goods that could provide enough positive well-being to outweigh their suffering.

## Conclusions

Our aim in this paper has been to reflect on whether it would be in the interests of an infant like Esther, who has a severe neurological disorder and reduced life expectancy, to undergo life-prolonging major surgery in the neonatal period. Other papers on this topic have focused on whether children like Esther would suffer, in addition to what it means for them to suffer. Our starting point is slightly different: we contend that the answer to this question depends on whether she would have a positive balance of well-being in her life overall.

To help answer this latter question we have isolated three relevant elements to the case, assessing the impact on Esther’s well-being of (1) having a short life, (2) having profound cognitive impairment, and (3) having (potentially) incomplete relief of physical suffering.

We argued first that positive well-being may be relatively limited in children who die in infancy or very early childhood. Such children may have lives worth living, yet the reduced components of both objective and subjective well-being may mean that the negatives more easily outweigh the positives in their lives. There would be negative net well-being, for example, in the lives of infants who die after a long stay in the neonatal intensive care unit associated with multiple painful procedures.

We did not explore whether this phenomenon of limited positive well-being extends beyond infancy, but rather identified the particular importance of the phenomenon for children whose cognitive capacities remain very limited. We noted that for children like Esther, the objective elements of well-being are highly likely to be attenuated. We separately explored the potential impact of profound cognitive impairment on subjective well-being, hypothesising that at very low levels of consciousness there may be the possibility of negative experiences but no (or minimal) positive experiences—a scenario we referred to as the dyshedonia effect.

Finally, if Esther were to undergo surgery, or experience pain or distress from another source, we outlined four reasons why medical treatment may incompletely relieve her suffering. First, it may be difficult for caregivers to reliably detect when an infant or child with profound cognitive impairment is experiencing pain or distress. Second, there are serious limitations to scientific knowledge relating to analgesia in this population. Third, treatment of pain may in some cases reduce consciousness to a degree that causes dyshedonia. Finally, the desire to avoid side effects from sedatives and analgesics (e.g., reduced consciousness, reduced respiratory drive) may lead professionals or parents to opt for relative undertreatment of pain.

We have not sought to directly address in this paper what should happen in Esther’s case. There are important further questions about the extent of parental discretion in the treatment of children and other relevant considerations regarding the use of limited health-care resources to provide treatments for infants like Esther. It is obviously extremely difficult to evaluate what would be in a child’s best interests, and in some cases there may be reasonable disagreement about the right course of action.

However, we maintain that the factors that we have discussed in this paper are important to consider. Where a child has a short life expectancy, profound cognitive impairment, and the potential to experience unrelieved suffering, life-prolonging therapy may not be in her best interests—and indeed may do her harm.

## Footnotes

1 The case of baby Esther is presented in Tyler Tate’s introduction to this *Theoretical Medicine and Bioethics* special issue on paediatric suffering [1].

2 In a cohort of surviving children with lissencephaly, the majority had a developmental age of one month or less [2]. Long-term survival of children with neurological impairment may depend on the extent of provision of life-prolonging treatment. Published data on survival are often difficult to use because of the problem of self-fulfilling prophecies [3].

3 The arguments about well-being presented in this paper are completely separate from questions about the moral status of foetuses, infants, or those with profound impairment. A reduction in well-being (even a conclusion that well-being is absent or negative) does not mean that an individual lacks moral status. Conclusions about well-being might have implications for how such an individual should be treated, but these implications will depend on the relevant account of moral status one adopts.

4 Hedonic theories focus on happiness, as well as avoidance of pain. For this paper, we largely set aside a third set of theories, desire- or preference-based theories, since we suspect that complex desires are largely absent in the context of profound cognitive impairment. A child or infant may have simple desires, but these will likely overlap substantially with hedonic considerations—for example, a desire for warmth, food, comfort, or proximity of care.

5 Imagine, abstractly, that a child lives for just one year and that a year of happiness has a value of 100 units. If the child could live for another year of happiness but does not, we should note that the value of her life is not increased by 100 units. However, it would be a mistake to think that the value of the child’s life is decreased by 100 units because she dies prematurely. That would imply that a year of life has, all things considered, zero value.

6 One immediate difficulty with this question is the non-identity problem and comparisons of never-existence with existence. It is beyond the scope of this paper to address that issue in any depth. However, even if it is problematic to claim that it would have been better for you never to have existed (since then there would have been no you to be better off), most accept that it can be bad to be brought into existence with overall negative well-being [8, p. 391].

7 Foetuses potentially have a developing relationship with their mother (and even other members of the family).

8 Some evidence suggests that foetuses may actually be in a state akin to sleep because of the combined influence of substances like adenosine, steroids, and prostaglandins, as well as the influence of relative hypoxia [10]. The sleep analogy does not depend on foetuses’ actually being asleep—though obviously if this is the case, the analogy is particularly apt.

9 Joshua Shepherd has argued that one distinguishing characteristic of disabilities that reduce well-being (as opposed to having a neutral effect on well-being) is the loss of control over one’s situation [18].

10 Whether or not Esther has the capacity for deep interpersonal relationships will depend on the severity of her cognitive impairment. In a Dutch cohort study of children with lissencephaly, one-third of the subjects were so impaired that they made no eye contact or purposeful movements [2]. If she is so profoundly impaired that she lacks ‘relational potential’ [20], including the capacity to recognise and interact with others, then the objective value afforded by deep interpersonal relationships may also be absent. In that case, as one of us has argued elsewhere, there may be little or no prudential value to her life [21].

11 For further discussion of the relevance of profound cognitive impairment to objective well-being, see [5, 22].

12 We describe this as a paradox because most people would intuitively regard an individual with more severe cognitive decline to be worse off. Yet it may actually be better (from the point of view of the individual’s hedonic experience) to have moderate or severe rather than mild dementia. Objective accounts of well-being would not necessarily reach this conclusion, since they are likely to identify overall reductions in well-being with cognitive decline. On such a view, a year of life with moderate or severe dementia would be much worse than a year of life with mild dementia—even if the individual would be happier in the former state.

13 It is sometimes claimed, for example, that children with trisomy 21 (Down syndrome) are happy—perhaps even more happy than cognitively normal children. There is some evidence that a high proportion of people with Down syndrome report being happy and have a positive self-perception [23]. It is difficult to know whether this trend is specific to the behavioural phenotype of trisomy 21. We have not been able to identify studies comparing self-reported well-being in children with other causes of similar intellectual disability or comparisons to those with milder or more severe cognitive disability. The evidence is also somewhat mixed. One systematic review found rates of depression in people with Down syndrome (5–13%) that were higher than in people with other forms of intellectual disability [24].

14 Note that such disability is considerably more severe than Down syndrome. The average mental age of individuals with trisomy 21 is often said to be eight to nine years. The arguments in this section do not apply to disabilities of this degree.

15 We acknowledge that our description here is an oversimplification. Pharmacological general anaesthesia involves a complex mix of effects: sedation/hypnosis, amnesia, analgesia, and muscle relaxation [26].

16 This is perhaps excluding those positive physical sensations caused as a side effect of the anaesthetic drugs.

17 The term ‘dyshedonia’ has sometimes been used in the context of psychiatric illness to refer to disordered perception of pleasure—for example, an inability to experience pleasure or feelings of distress in response to ordinarily pleasurable stimuli. Here we use the term to refer to a disordered balance of positive and negative experiences.

18 One potential argument against the dyshedonia hypothesis is that this might imply that nonhuman creatures with very low levels of consciousness, such as molluscs or crustaceans, have net negative well-being (i.e., lives that are not worth living). An analysis of sentience or well-being in such animals is beyond the scope of this paper. However, a potential response to this argument might be to accept that overall well-being is negative for animals with minimal levels of consciousness. Alternatively, there might be evolutionary reasons for such creatures to have some positive sensations (e.g., taste, relief of hunger) [30]. Such evolutionary reasons would not necessarily apply in the very unnatural state of humans with low levels of consciousness. Finally, one counterbalancing positive for nonhuman animals with low levels of consciousness may be a form of agency—the ability to move, seek nutrients, or reproduce. The good of agency, whether understood objectively or subjectively, does not seem to be present in humans in a minimally conscious state. We are grateful to Tyler Tate for this suggestion.

19 There is evidence to suggest that suffering goes under-recognised in both adults [31] and infants [32]. A number of tools for assessing pain in infants have been developed, which rely on a combination of objective measures, such as heart rate and blood pressure, and subjective observations of grimacing, crying, and overall demeanour [33]. However, a variety of methods and scales are used, with no agreed-upon universal procedure for measuring pain and distress. Sedation, which is often used in intensive care, makes assessment of pain and discomfort even more difficult by masking the few behavioural signs assessors have to rely on. Better measures of physical pain in infants may come in the future through the prospect of direct measurement of nociceptive (pain-related) brain activity [32]. However, none of these tools can offer insight into the subjective experience of pain and suffering in infants.

20 This ‘silent suffering’ problem may be relevant not only for physical pain or discomfort, but for other negative emotional states as well. Adults can experience fear, anxiety, and distress while sedated or anaesthetised [27].

21 Similar uncertainties potentially affect treatment of pain in children with severe neurological impairments.

22 The doctrine of double effect is often used to justify the permissibility of an action that causes a serious harm, such as the death of a human being, as a side effect of promoting some good end, such as the relief of suffering.
